# Minimal access versus open spinal surgery in treating painful spine metastasis: a systematic review

**DOI:** 10.1186/s12957-015-0468-y

**Published:** 2015-02-21

**Authors:** Zuozhang Yang, Yihao Yang, Ya Zhang, Zhaoxin Zhang, Yanjin Chen, Yan Shen, Lei Han, Da Xu, Hongpu Sun

**Affiliations:** Bone and Soft Tissue Tumors Research Center of Yunnan Province, the Third Affiliated Hospital of Kunming Medical University (Tumor Hospital of Yunnan Province), Kunming, Yunnan 650118 PR China

**Keywords:** Minimal access, Open spinal surgery, Spine metastasis, Systematic review

## Abstract

**Study design:**

The study design of this paper is a systematic review of literature published in the recent 10 years.

**Objective:**

It is the objective of this paper to compare the clinical efficacy and safety of minimal access (MIS) spinal surgery and open spinal surgery for treating painful spine metastasis.

**Methods:**

Two research questions below were determined through a consensus among a panel of spine experts. A systematic review of literature on spinal surgery was conducted by searching PubMed with a combination of keywords including “metastatic”, “metastasis”, “metastases”, “spinal”, and “spine”. Independent reviewers selected the articles for analysis after screening the titles, abstracts, and full texts, then extracted data and graded the quality of each paper according to the Grading of Recommendations, Assessment, Development, and Evaluation (GRADE) criteria. Specific clinical questions were as follows:

1. In patients with spine metastatic disease, what is the impact of different surgical approaches (MIS versus open) on pain relief and functional outcome?
2. In patients with metastatic disease, what is the impact of different surgical approaches (MIS versus open) on local recurrence, survive rate, and complication?

**Results:**

A total of 1,076 abstracts were identified using various keywords. 5 prospective (level II) and 12 retrospective articles (level III) were eligible for inclusion, involving a total of 979 cases of spine metastasis. There were 345 cases in 8 studies regarding the clinical evaluation of MIS spinal surgery and 634 cases in 9 studies regarding the clinical evaluation of open spinal surgery for spine metastasis.

**Conclusion:**

Both open spinal surgery and MIS seem to achieve the improvement of pain and neurological dysfunction through decompression and stabilization for patients with spine metastasis, but open surgery may involve more major complications with a trend of lower survival rates and higher recurrence rates compared to MIS.

## Review

### Introduction

About 10% of cancer patients develop metastases in the spine [1]. The most common site of metastasis in the spine is the thoracic spine (50% to 60% of all metastases), followed by the lumbar (30% to 35%) and cervical spines (10% to 15%) [2-4]. Vertebral metastasis is one of the most serious life-threatening diseases [5]. About one third of patients with spinal metastases become symptomatic, which means intractable pain, neurological deficits, and/or biomechanical instability requiring surgical treatment [3,6].

These nonsurgical methods, such as chemotherapy, radiotherapy, and hormonal therapy, were commonly used for the management of vertebral metastatic disease, which have been proven to be effective in halting the osteolytic process and reversing the neurological compromise [7]. However, these modalities are unable to provide stability to an instable spinal column and also unable to be expected to relieve pain and spinal cord compression. In these cases, surgery is the best method for the resolution of intractable pain, neurological compromise, and overt or impending spinal instability in patients with spinal metastases. The primary goal of the surgery is to improve patients’ quality of life by providing pain relief, maintaining or improving neurological function, and restoring the structural integrity of the spinal column [8].

Stabilization of the spine is often necessary as extensive lesions may cause spinal instability by erosion of the normal bony structures. The most efficacious therapy for restoring spinal instability is reconstructive surgical intervention. Unfortunately, lots of patients are not considered as candidates for conventional surgery intervention due to neoplasm-associated comorbidities such as malnourishment and a weak immune system that make amounts of surgical procedures unfeasible [9]. However, these patients can be managed with vertebral augmentation, since it can provide some degree of restabilization [10].

Vertebroplasty or kyphoplasty, which involves the percutaneous injection of polymethyl methacrylate (PMMA) bone cement into a collapsed vertebral body, is a currently available minimally invasive spine surgery for palliative treatment. Poor surgical candidates with disabling pain secondary to a pathologic thoracic or lumbar vertebral body fracture without epidural compression are ideal candidates for these procedures [11]. The two procedures have been shown to relieve pain effectively to improve the quality of life, and they can be used as effective palliative treatment even for patients whose general condition is quite poor, with decreased pain, less blood loss, and shorter hospital stays [12]. On the other hand, their putative benefits with regard to spinal stability and neurological function, as well as their risks affecting their overall survival, have not yet been adequately documented by clinical studies because of limitations such as insufficient spinal cord decompression and stabilizing the vertebral column.

Despite numerous reports on open spinal surgery in treating painful spine metastasis, there exist no randomized controlled comparisons of clinical efficacy and safety between open and minimal access (MIS) procedures. In addition, due to the heterogeneity of study designs, inconsistent reporting of complications, and the use of different grading scales for pain and functional outcomes, it was not possible to perform a meta-analysis using the prospective and retrospective studies. Therefore, we endeavored to perform a quantitative systematic review of the current literature published in the recent 10 years to evaluate the clinical efficacy between MIS and open spinal surgery in patients with spinal metastases. A secondary aim was to compare complication rates between MIS and open spinal surgery.

### Materials and methods

Two clinically relevant questions below were determined through a consensus among a panel of spine oncology experts (the Spine Oncology Study Group), and a systematic review of related literature published in the recent 10 years was conducted using PubMed. Specific clinical questions were as follows:

1. In patients with metastatic disease, what is the impact of different surgical approaches (MIS versus open) on alterations of pain and neurologic function?
2. In patients with metastatic disease, what is the impact of different surgical approaches (MIS versus open) on local recurrence, survive rate, and complication?

#### Search criteria

We used the search terms that included “spin*”, “metasta*”, and “surg*” to searched literature from PubMed. The following terms would be contained: “metastatic”, “metastasis”, “metastases”, “spinal”, “spine”, “surgery”, and “surgical”.

Criteria for possible inclusion are as follows: 1) articles published in the recent 10 years, 2) all articles in English or with an English translation, 3) articles with 20 or more subjects, 4) adult age group (18 years and older), 5) articles describing surgical treatment of spinal metastatic cancer, and 6) articles evaluating the alterations of pain and neurologic function postoperatively. Exclusion criteria include the following: 1) primary tumors, 2) intradural tumors, 3) pediatric age group, 4) articles with fewer than 20 subjects, and 5) articles with nonhomogeneous pathology (e.g., trauma and primary tumors in the same series).

Studies were reviewed using a standardized data collection form. The type of study (prospective or retrospective) was noted. Data including surgery technique, the total number of patients, and the type of tumors were totally collected. The methods of pre- and postoperative clinical evaluations with respect to pain and functional outcome were also recorded. All temporary and permanent complications were collected, including major and minor complications. To avoid duplicate records of patients’ data, each group or institution was limited to one study in the systematic review. Some authors were contacted directly to clarify certain aspects of their studies.

The quality of evidence for each article was evaluated as high, moderate, low, or very low. The results of the systematic review and ratings of the evidence for each article were determined by a multidisciplinary, international group of spine oncology surgeons, oncologists, and methodologists (Spine Oncology Study Group). The group then went through a consensus-based decision-making process using a modified Delphi technique to arrive at treatment recommendations related to the key clinical questions. This process and the strength of the recommendation were based on the Grading of Recommendations, Assessment, Development, and Evaluation (GRADE) method [13,14]. These articles were evaluated independently by the authors according to the GRADE criteria.

### Result

**Figure 1:**
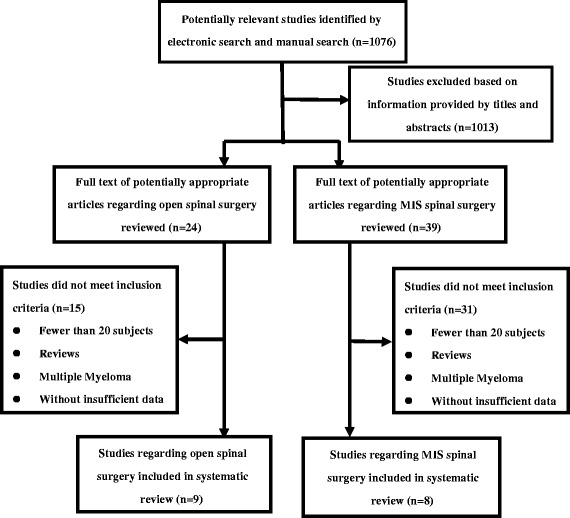
**Flow diagram showing identification of studies included in the review.**

A total of 1,076 abstracts were identified using various keywords. All abstracts were screened, and 1,013 articles were excluded as obviously unrelated. The full texts of 63 papers were screened and 17 papers were identified to meet the inclusion criteria, including 5 prospective (level II) [15-19] and 12 retrospective articles (level III) [20-31] involving 979 cases of spine metastasis. The details of article selection were presented in Figure 1. There were 345 cases in 8 studies regarding the clinical evaluation of MIS spinal surgery and 634 cases in 9 studies regarding the clinical evaluation of open spinal surgery for spine metastasis (Table 1). In one study, the primary cancer was breast cancer, whereas in other studies were a mixed group such as lung cancer, prostate cancer, and colon cancer.

**Table 1:** **Characteristics of included studies**

**Study**	**Year**	**Type of study**	**Quality**	**Number of Patients**	**Mean age**	**Procedure**	**Primary**	**Outcome**	**Follow-up**
Minimal									
Pizzoli *et al.* [20]	2009	Retrospective	Very low	39	NA	PVP with PMMA	Mixed	VAS, mobility, analgesic use	NA
Chen *et al.* [21]	2009	Retrospective	Very low	31	67	PVP with PMMA	Mixed	VAS, Karnofsky scale	12 months
Qian *et a*l. [22]	2011	Retrospective	Very low	48	68.5	Kyphoplasty	Mixed	VAS, ODI, SF-36, vertebral body height variation	24 months
Farrokhi *et al.* [15]	2012	Prospective	Very low	25	53.5	PVP with PMMA	Mixed	VAS	NA
Tseng *et al.* [23]	2008	Retrospective	Very low	57	65.18	PVP with PMMA	Mixed	VAS, amounts of nonnarcotic and narcotic analgesic	6 months
Yang *et al.* [16]	2009	Prospective	Very low	40	57.63	PVP with PMMA	Mixed	VAS, KPS	1 year
Pflugmacher *et al.* [17]	2008	Prospective	Very low	65	66	Balloon kyphoplasty	Mixed	VAS, ODI	24 months
Anselmetti *et al.* [18]	2013	Prospective	Very low	40	66.8	PVP with PEEK	Mixed	VAS, ODI	10 months
Open									
Holman *et al.* [24]	2005	Retrospective	Very low	139	55	Posterior/anterior/combined tumor resection, decompression, instrumentation, fusion	Mixed	VAS, Frankel grades	12 months
Chong *et al.* [25]	2012	Retrospective	Very low	105	58.3	Single-stage posterior decompression and stabilization	Mixed	VAS, Frankel grade, KPS	48 months
Eid *et al.* [26]	2011	Retrospective	Very low	45	53	Circumferential decompression and fusion by means of PTA	Mixed	VAS, ASIA grades	13 months
Liang *et al.* [27]	2013	Retrospective	Very low	92	NA	Posterior/anterior/combined tumor resection, decompression, instrumentation, fusion	Mixed	VAS, Frankel grades, Karnofsky score, Tokuhashi scores	22 months
Wonik Cho *et al.* [28]	2012	Retrospective	Very low	46	56.4	ACF, ACF and PF, DLF, DL	Mixed	VAS, JOAS, Tomita score	39 months
Walter *et al.* [29]	2012	Retrospective	Very low	57	58.6	Posterolateral approach for decompression combined with posterior instrumentation	Mixed	VAS, Frankel grades, ECOG grades, TPS	46 months
Dae-Chul Cho *et al.* [30]	2009	Retrospective	Very low	21	56.6	Posterolateral transpedicular approach with posterior instrumentation	Mixed	VAS, Frankel grades, and ECOG grades	13 months
Shehadi *et al.* [31]	2007	Retrospective	Very low	87	53	Posterior/anterior/combined tumor resection, decompression, instrumentation, fusion	Breast cancer	VAS, Frankel grades	13 months
Street *et al.* [19]	2007	Prospective	Very low	42	56.04	Single-stage posterolateral vertebrectomy	Mixed	VAS, ECOG grading	NA

*PVP* percutaneous vertebroplasty, *PMMA* polymethyl methacrylate, *PEEK* polyetheretherketone, *VAS* visual analog scale, *ODI* Oswestry Disability Index, *JOAS* Japanese Orthopaedic Association Score, *KPS* Karnofsky performance scores, *ASIA* American Spinal Injury Association, *ECOG* Eastern Cooperative Oncology Group, *PTA* posterolateral transpedicular approach, *TPS* Tomita’s prognostic score, *ACF* anterior corpectomy and fusion, *ACF and PF* ACF and posterior fixation, *DLF* decompressive laminectomy posterior fixation, *DL* decompressive laminectomy, *NA* not available, *SF-36* Short Form 36 Physical Function.

Among these articles regarding open spinal surgery, three studies were performed with several kinds of approaches such as posterior, anterior, and combined approach to remove tumors in spines. Studies regarding MIS spinal surgery were performed primarily with vertebroplasty whereas two studies [22,17] were performed with kyphoplasty.

Pain alleviation was crucially important for the therapy of spine metastases to improve quality of life in patients. A visual analog scale was available for each study to evaluate the effect of pain relief in patients with spine metastases. In all, one study did not display the visual analog scale (VAS) score in detail [26], and prospective studies had especially detailed pre- and postoperative VAS score. It was found that both MIS and open spinal surgery were efficient in pain alleviation, and most of the studies reported statistically significant improvement (Table 2).Given the high rates of pain alleviation, the results suggest that both MIS and open spinal surgery are efficacious in respect to pain alleviation through decompression and stabilization.

**Table 2:** **VAS scores in included studies**

**Study**	**Pre**	**Post**	**P value**	**Follow-up**	**Pain relief rate**
Minimal					
Pizzoli *et al.* [20]	8.62 (±0.71)	2.84 (±1.36)	NA	24 h	98%
Chen *et al.* [21]	8.9 (±0.93)	2.6 (±1.71)	*P* < 0.001	NA	94%
Qian *et al*. [22]	7.4 (±2.1)	3.8 ( ±1.6)	*P* < 0.001	24 h	NA
		3.2 (±1.0)	*P* < 0.001	6 months	NA
Farrokhi *et al.* [15]	8.23 (NA)	2.12 (NA)	*P* ≤ 0.05	24 h	NA
		1 (NA)	*P* ≤ 0.05	2 months	NA
Tseng *et al.* [23]	8.1 (±0.67)	3.8 (±1.9)	*P* < 0.015	24 h	NA
		2.8 (±2.0)	*P* < 0.001	6 months	NA
Yang *et al.* [16]	8.78 (±0.54)	5.41 (±0.94)	*P* = 0.032	6 months	95.00%
Pflugmacher *et al.* [17]	8.3 (±15)	3.3 (±9)	*P* < 0.0001	3 months	NA
Anselmetti *et al.* [18]	10 (±1)	1 (±0.75)	*P* < 0.001	1 month	100.00%
Open					
Holman *et al.* [24]	7 (NA)	2 (NA)	*P* < 0.001	1 month	94%
Chong *et al.* [25]	6.5 (±1.7)	3.4 (±1.6)	NA	2 weeks	NA
Eid *et al.* [26]	NA	NA	NA	1 month	96%
Liang *et al.* [27]	6 (NA)	2 (NA)	*P* < 0.001	12 months	90%
Wonik Cho *et al.* [28]	7.86 (±1.05)	4.48 (±2.09)	*P* = 0.001	NA	NA
Walter *et al.* [29]	6.9 (±1.6)	3.1 (±1.0)	*P* < 0.001	1 month	87.70%
Dae-Chul Cho *et al.* [30]	6.82 (±2.13)	3.61 (±1.01)	NA	1 month	NA
Shehadi *et al.* [31]	6 (NA)	2 (NA)	*P* < 0.001	1 month	NA
Street *et al.* [19]	7.94 (NA)	4.3 (NA)	*P* < 0.001	NA	NA

Each study assessed postoperative neurologic function, and multiple methods for evaluating neurologic function were included, such as the Eastern Cooperative Oncology Group performance scale, the Oswestry Disability Index, the Frankel scale, the Karnofsky scale and American Spinal Injury Association grades, the Short Form 36 Physical Function, the Japanese Orthopaedic Association Score, and the Karnofsky performance scores. The neurologic function was not assessed in two studies (Farrokhi *et al.* [15] and Tseng *et al.* [23]) regarding MIS surgery. Only nine studies provided pre- and postoperative neurologic function score, and neurologic function was statistically significantly improved (Table 3). Other studies without detailed data also showed that MIS and open spinal surgery were successful in improving physical function.

**Table 3:** **Neurologic function evaluation in included studies**

**Study**	**Method**	**Scale best to worst**	**Functional outcome**	***P*** **value**
Minimal				
Pizzoli *et al.* [20]	Mobility	1 to 4	Preoperative: 3.25 ± 0.59, postoperative: 1.24 ± 0.64	*P* < 0.001
Chen *et al.* [21]	Karnofsky scale	100 to 0	Preoperative: 50 ± 10.65, postoperative: 70 ± 3.59	NA
Qian *et al.* [22]	ODI	0 to 100	Preoperative: 71.5 ± 16.7, postoperative: 29.5 ± 10.2	*P* < 0.001
	SF-36	100 to 0	Preoperative: 34.3 ± 10.8, postoperative: 54.5 ± 10.5	*P* < 0.05
Yang *et al.* [16]	Karnofsky scale	100 to 0	Preoperative: 69.4 ± 8.3, postoperative: 80.3 ± 7.2	*P* = 0.002
Pflugmacher *et al.* [17]	ODI	0 to 100	Preoperative: 81 ± 8, postoperative: 39 ± 7	*P* < 0.0001
Anselmetti *et al.* [18]	ODI	0 to 100	Preoperative: 82.2, postoperative: 4.1	*P* < 0.001
Open				
Holman *et al.* [24]	Frankel grade	E to A	46/112 (41%) improved at least one Frankel grade, 20/112 (18%) regained ambulation, seven (5%) worsened	*P* < 0.05
Chong *et al.* [25]	Frankel grade	E to A	21/105 (20%) improved at least one Frankel grade, 21/105 (48%) regained ambulation, 6/105 (5.7%) worsened	*P* < 0.05
Eid *et al.* [26]	ASIA grade	E to A	23/45 (51%) improved one or more grades, 6/45 (20%) retained their preoperative grade, one (3%) experienced worsening	*P* > 0.05
Liang *et al.* [27]	Karnofsky scores	100 to 0	The median postoperative Karnofsky scores increased from 60 (range, 40 to 80) to 70 (range, 0 to 80)	*P* < 0.001
	Frankel grade	E to A	78% improved 1.2 grades at average	NA
Wonik Cho *et al.* [28]	JOAS	10 to 0	Preoperative: 13.11 ± 2.75, postoperative: 15.17 ± 2.09	*P* = 0.001
Walter *et al.* [29]	Frankel grade	E to A	13 (22.8%) patients improved, 43 (75.5%) had a stable neurological status, one single patient (1.8%) experienced worsening	NA
	ECOG grade	0 to 5	Preoperative: 2.0 ± 1.1, postoperative: 1.7 ± 1.3	*P* < 0.05
Dae-Chul Cho *et al.* [30]	Frankel grade	E to A	7/22 (33.3%) improved, 14/21 (66.7%) had a stable neurological status	NA
	ECOG grade	0 to 5	8 had an improved ECOG grade, and 12 showed no change, 1 experienced worsening	NA
Shehadi *et al.* [31]	Frankel grade	E to A	85% maintained or improved their Frankel scores	NA
Street *et al.* [19]	ECOG grade	0 to 5	Preoperative: 2.5 ± 1.0, postoperative: 1.6 ± 0.75	NA

*ODI* Oswestry Disability Index, Japanese Orthopaedic Association Score, *ECOG* Eastern Cooperative Oncology Group, *NA* not available, *SF-36* Short Form 36 Physical Function.

It is reported that decreased complication rates are considered to be one of the advantages of MIS spinal surgery. This was confirmed by the evidences we provided in this systematic review. There are no major complications for MIS spinal surgery except in two studies [20,23], whereas six studies regarding open spinal surgery reported major complications with a trend of lower survive rates and higher recurrence rates (Table 4).

**Table 4:** **Serious complications in included studies (excluding deaths)**

**Study**	**Recurrence**	**Survival**	**Complications**
Minimal			
Pizzoli *et al.* [20]	5.1%	NA	Three major complications (one pneumothorax and two symptomatic leakages) (2.8%), two minor complications (cement pulmonary embolism) (1.8%)
Chen *et al.* [21]	NA	74% at 6 months, 39% at 12 months	No major complication
Qian *et al.* [22]	No	81% at 2 years	No major complications, cement leakage (18.6%)
Farrokhi *et al.* [15]	NA	NA	Cement leakage (44%)
Tseng *et al.* [23]	NA	Two patients died during hospitalization	Cement extravasation (17.9% minor extravasation, 3.9% major extravasation)(21.8%)
Yang *et al.* [16]	No	80.0% at 1 year	Seven paravertebral cement leakage (17.5%)
Pflugmacher *et al.* [17]		80.0% at 1 year, 66% at 2 years	Cement leakage (12.1%), adjacent incident fracture (8%)
Anselmetti *et al.* [18]	20%	85% at 3 months	Cement leakage (16.3%)
Open			
Holman *et al.* [24]	8%	Mean survival was 14.8 months, 67% at 0.5 months, 54% at 1 month, 23% at 5 years	Major complications (18%) and minor complications (21%)
Chong *et al.* [25]	NA	Median survival was 6.0 months, 34% at 1 year, 14% at 2 years survival rates	Surgical complications occurred in patients (10%), no mechanical failure
Eid *et al.* [26]	No	Mean survival was 13 months	Unstable (15.5%), wound infection (15.5%), hematoma (4%) and deep vein thrombosis (2%)
Liang *et al.* [27]	NA	The median survival was 15 months, 61% at 1 year, and 35% at 3 years	Major complications (23%)
Wonik Cho *et al.* [28]	39.10%	Mean survival was 11.82 months, 44.4% at 6 months, 35.6% at 12 months, and 19.0% at 24 months	Two operation site infection, two pneumonia, one esophageal fistula after anterior approach (10.9%)
Walter *et al.* [29]	1.70%	Mean survival was 11.4 months, 42.1% at 1 year	Superficial wound infections and one seroma (5.3%)
Dae-Chul Cho *et al.* [30]	14%	Mean survival was 8.9 months	One wound infection and one wound dehiscence (9.5% )
Shehadi *et al.* [31]	11.50%	Median survival was 21 months, 62% at 1 year, 33% at 3 years, and 24% at 5 years	Major complications by surgical approach (17%)
Street *et al.* [19]	2.30%	75% at 6 months and 50% at 12 months	Major complications (26%)

*NA* not available.

### Discussion

For most of the patients with spinal metastasis, the treatment is largely palliative and aims to achieve relief of pain and regain function, thus improving the quality of the life of the patients. Because of the immunocompromised status, poor nutrition, and comorbid medical conditions, many patients with spinal metastasis cannot tolerate the curative surgical methods. In recent years, more and more minimally invasive spinal interventions are reasonable alternatives to open spinal surgery for treating spinal metastatic tumors. These procedures can contribute to less soft tissue trauma, lower blood loss, and shorter hospitalization time. MIS spinal surgery rarely interferes with the adjuvant treatments. The overall morbidity is considerably lower in comparison to conventional spine surgery.

Despite this evolution, questions surrounding the effectiveness of MIS and its comparability to open spinal surgery in terms of pain and neurologic function remain unanswered. A direct comparison of clinical efficiency and safety between open and MIS spinal surgery for spinal metastasis has never been conducted. Given the lack of comparison studies, we aimed to compare the effect of pain alleviation and functional improvement between open and MIS spinal surgery by reviewing published studies in a quantitative manner. Our results suggest that open or MIS spinal surgery is likely to achieve the improvement in pain and neurological dysfunction for spinal metastasis, but open surgery seems to involve more major complications with a trend of lower survival rates and higher recurrence rates compared to MIS surgery.

There are few comparison studies between MIS and open spinal surgery. Huang *et al.* [32] performed a direct retrospective comparison of MOT, MBL, LOS, and CR for MIS versus open spinal surgery for thoracic spine metastasis, and no significant difference was found. However, the amount of patients requiring at least a 2-day admission in the open group was significantly larger than that of the MIS group (open: 88% versus MIS: 6.9%). If there is truth that there is no significant difference in the functional outcome between the MIS and open group, the possible reason is that the potential benefit of MIS is counteracted by the more complicated nature of patients with metastatic spine disease during operation [33]. Payer *et al.* [34] also demonstrated that mean blood loss, operative time, and complication rates in spinal tumor patients were higher than that of the fracture patients with anterior approach.

## Conclusions

In conclusion, we performed the systematic review based on literature published in the recent 10 years to possibly compare clinical efficiency and complication rate between open and MIS spinal surgery for spine metastasis and found that both open spinal surgery and MIS seem to achieve the improvement of pain and neurological dysfunction through decompression and stabilization, but open surgery may involve more major complications with a trend of lower survival rates and higher recurrence rates compared to MIS. However, it is necessary to perform a controlled study to compare the clinical efficiency between the two procedures for spine metastasis in the future.
